# Perioperative Anesthetic and Hormonal Considerations in a Patient With Anti-glutamic Acid Decarboxylase Encephalitis: A Case Report

**DOI:** 10.7759/cureus.92914

**Published:** 2025-09-22

**Authors:** Ian W McArdle, Haadi Malik, Sarah Dotson, Arpan Kohli

**Affiliations:** 1 Anesthesiology and Perioperative Medicine, West Virginia University School of Medicine, Morgantown, USA; 2 Anesthesiology, West Virginia University School of Medicine, Morgantown, USA; 3 Obstetrics and Gynecology, West Virginia University School of Medicine, Morgantown, USA

**Keywords:** anti-gad 65 encephalitis, anti-gad antibodies, gyn pathology, neuro anesthesia, neuro oncology

## Abstract

Glutamic acid decarboxylase (GAD) is vital for gamma-aminobutyric acid (GABA) synthesis. Auto-antibodies against GAD deplete GAD levels and subsequently GABA, resulting in a hyper-excitatory state. A 41-year-old woman with anti-GAD encephalitis underwent bilateral oophorectomy for endometriosis. Despite treatments, the patient experienced limited improvement. Suppression with Depo-Lupron and subsequent bilateral oophorectomy resulted in significant improvement. Post-oophorectomy, she experienced seizure-like episodes, suggesting medication's role in modulating neuroexcitability. The case underscores the interplay between anti-GAD antibodies and general anesthetics, highlighting the importance of understanding perioperative management of autoimmune encephalitis. Research is needed for an optimal approach to this patient population, emphasizing interdisciplinary collaboration.

## Introduction

Glutamic acid decarboxylase (GAD) is the rate-limiting step in the synthesis of the inhibitory neurotransmitter γ-aminobutyric acid (GABA) [1]. In the setting of autoantibodies against GAD, depleted GAD levels result in decreased GABA synthesis. This imbalance of inhibitory neurotransmitters results in a hyper-excitatory state, laying the foundation for hyper-excitatory clinical manifestations. Anti-GAD antibodies were first described in 1988 in a patient with stiff-person syndrome, epilepsy, and type 1 diabetes [2]. Anti-GAD antibodies have now been associated with multiple neurological syndromes, such as epilepsy, stiff-person syndrome, limbic encephalitis, and cerebellar ataxia [3,4].

These neurological symptoms are frequently linked to a personal or family history of autoimmunity. Approximately half of the patients will have a history of type 1 diabetes mellitus. Most patients will present with another form of systemic autoimmunity. A small percentage of these neurological syndromes can be associated with an underlying malignancy. Conditions more commonly associated with malignancy include cerebellar ataxia and limbic encephalitis. Screening for malignancy should be considered when common risk factors are present, such as older age and male gender [5].

Anti-GAD encephalitis is characterized by a more subacute or chronic symptom progression compared to other causes of encephalitis. It mainly presents as seizures, movement disorders, limb or trunk stiffness, muscle spasms, cognitive decline, slurred speech, and walking instability [4]. There is no standardized treatment, but patients have reported improvement with long-term immunotherapy including corticosteroids and immunoglobulins [4].

Anti-GAD-mediated neurological syndromes are uniquely influenced by both endocrine and anesthetic factors. Exacerbations have been associated with hormonal fluctuations, such as those occurring during the menstrual cycle, pregnancy, or in estrogen-dependent conditions like endometriosis [6,7]. Similarly, anesthetic agents that act on the GABAergic system, including benzodiazepines and propofol, can transiently restore inhibitory balance in patients with GABA deficiency [8-12]. These observations underscore the complex interplay between immunology, endocrinology, and anesthetic pharmacology in this patient population and highlight the importance of interdisciplinary awareness during perioperative care.

We present a case of a 41-year-old woman with anti-GAD encephalitis presenting for bilateral oophorectomy for the treatment of endometriosis, which appeared to be exacerbating her anti-GAD-mediated symptoms.

## Case presentation

At age 32, this patient presented with headaches, ataxia, tremors, and cognitive difficulties. Her symptoms were exacerbated by stress, pregnancy, and her menstrual cycle. Anti-GAD antibodies were identified through laboratory work with a value of 0.24 at the time of diagnosis (reference < 0.02), and she was presumed to have a diagnosis of GAD encephalitis. She had limited improvement despite trials of high-dose steroids, intravenous immunoglobulin, and plasmapheresis. Rituximab was initiated, resulting in immediate improvement; however, she reacted adversely with decreasing CD19 counts. Rituximab treatment was stopped, which resulted in the normalization of her CD19 counts; however, this was accompanied by the return of her symptoms. One year later, a malignant scalp tumor was removed, with the pathology raising concerns for melanoma (v-raf murine sarcoma viral oncogene homolog B1 v600 positive nevus). An MRI was obtained at this time and revealed no abnormal intracranial processes.

Five years later, she sought consultation with a gynecologist due to progressively heavy, irregular menses and severe dysmenorrhea, unresponsive to conservative management with oral contraceptives, Depo-Provera, and a levonorgestrel intrauterine device. She described heavier bleeding when having an “anti-GAD flare” and requested hysterectomy for definitive management of her bleeding and dysmenorrhea. In March 2020, she underwent total laparoscopic hysterectomy and bilateral salpingectomy, as well as fulguration of Stage II endometriosis, which improved her symptoms of pelvic pain for 6-7 months. She disclosed a history of bradycardia during previous general anesthesia and heightened tremors/myoclonic jerks during the emergence from anesthesia. The patient’s neurologist recommended prophylactic use of benzodiazepines peri-operatively. She was given 2 mg intravenous midazolam and 40 mg oral aprepitant pre-operatively. The case was completed with an induction consisting of propofol, lidocaine, fentanyl, and rocuronium. Maintenance of anesthesia was with sevoflurane. An additional 1 mg intravenous midazolam was administered following extubation. The patient did well post-operatively and was discharged from the PACU.

This procedure improved her pelvic pain symptoms for a few months; however, the symptoms returned and she requested a bilateral oophorectomy for definitive management of her endometriosis. Anti-GAD antibody levels were obtained at her six-month follow-up visit and significantly decreased to a level of 0.08. She initiated Depo-Provera for suppression for six months which was ineffective, and she reported worsening neurologic symptoms of headache, fatigue, and tremors associated with flares in her pelvic pain. She was then started on Depo-Lupron monthly for three months, which drastically improved both her pelvic pain and endometriosis, and she was subsequently transitioned to oral elagolix (a gonadotropin hormone-releasing hormone agonist) with a plan to continue for two years with Aygestin add-back therapy. After 16 months, she reported decreased effectiveness in controlling her endometriosis pain and neurologic symptoms and requested bilateral oophorectomy for definitive treatment of endometriosis.

The patient underwent laparoscopic bilateral oophorectomy in January 2023. She did not receive preoperative midazolam for anxiolysis or pretreatment of her previously reported myoclonic jerking. The case was completed with an induction consisting of propofol, lidocaine, fentanyl, and rocuronium. Maintenance was again with sevoflurane. After emergence, she had multiple episodes of seizure-like activity with myoclonic jerking shortly after arrival to the recovery area. Midazolam was given for presumed seizures; however, myoclonic jerking would again occur after the effects of the medication decreased.

The patient was admitted overnight without further episodes of myoclonic jerking. Neurology was consulted and recommended against a routine EEG, noting that midazolam would interfere with the results. As the patient had returned to baseline, the EEG was deemed unnecessary. She was discharged the following day after confirming she had returned to her baseline status. At six months post-surgery, she reported no pelvic pain and complete resolution of her neurological symptoms with return of anti-GAD antibody levels within the normal reference range.

## Discussion

Central nervous system hyperexcitability presents with various phenotypic manifestations, ranging from stiffness and rigidity to myoclonus. Within this spectrum, GAD antibody-associated encephalitis has a notable predilection for an association with malignancies, surpassing other anti-GAD syndromes in this regard. The underlying malignancies implicated in these neuroimmunological disorders include small cell lung cancer, thymoma, breast cancer, and melanoma [5]. Although this patient did have a previous melanoma excision, her GAD antibody-associated encephalitis was most likely immunologic in nature because of the lack of cessation after removal of her melanoma.

The patient presented with a diverse range of symptoms, including headaches, ataxia, tremors, myoclonic jerking, and vision changes. These symptoms were exacerbated with stress and fatigue, and despite various treatments, there was minimal improvement. Notably, she experienced symptom worsening linked to her heavy menstrual cycles, likely due to the pro-inflammatory cytokine release associated with endometriosis. Suppression of endometriosis was associated with improvement in neurologic symptoms, with bilateral oophorectomy having the most profound effect. Importantly, while hysterectomy resolved her abnormal bleeding, it did not adequately suppress the estrogen-driven activity of her endometriosis. Endometriosis is an estrogen-dependent disease, and hysterectomy alone is often insufficient for definitive management unless accompanied by bilateral oophorectomy [6,7]. This distinction explains why her neurological and pain symptoms persisted after hysterectomy but improved following bilateral oophorectomy.

Following laparoscopic bilateral oophorectomy, she encountered seizure-like episodes accompanied by myoclonic jerking. Interestingly, following hysterectomy, she did not encounter similar issues. The distinguishing factors were the administration of aprepitant and midazolam. Notably, aprepitant has a dose-dependent inhibitory effect on cytochrome P450 3A4, potentially leading to an increased duration of action of midazolam [8]. Furthermore, the patient received 2 mg of preoperative midazolam along with 1 mg prophylactically after extubation following the hysterectomy. The additional benzodiazepine may have provided a protective effect against post-emergence hyperexcitability by bridging the transition between anesthetic agents and endogenous GABAergic deficiency. Benzodiazepines are positive allosteric modulators of the GABA-A receptor, enhancing inhibitory neurotransmission, and have been reported to be effective in suppressing rigidity, myoclonus, and seizures in anti-GAD-mediated disorders [9-12]. The absence of pre- and postoperative midazolam during her oophorectomy may therefore have unmasked her vulnerability to postoperative excitatory symptoms.

This opens the door for consideration of GABAergic medications to help bridge the gap of heightened neuroexcitability not only in anti-GAD antibody conditions but also in other hyperexcitable pathologies. There have been case reports with anesthetic recommendations for stiff-person syndrome, an anti-GAD antibody-associated disorder; however, there are fewer than 200 reported cases worldwide [9-11]. The potential for rigidity and the need for postoperative mechanical ventilation are considerations in the event of an exacerbation, possibly triggered by the stress of extubation. The anesthetic approach otherwise follows a standard protocol, including preoperative administration of benzodiazepines, induction with propofol and rocuronium, maintenance with volatile anesthetics, and the use of opioids as necessary [9-12]. More recent studies suggest that dexmedetomidine may be beneficial during emergence to minimize agitation and stress responses [12].

## Conclusions

This case highlights the complex interplay between anti-GAD antibodies, hormonal influences, and anesthetic management. The patient’s neurological symptoms appeared to improve following suppression of estrogen production, with bilateral oophorectomy providing the most durable benefit. Perioperatively, seizure-like episodes occurred after surgery when benzodiazepine coverage was not provided, suggesting that GABAergic support may be beneficial in this patient population. While interactions between aprepitant and midazolam may have contributed to her differing postoperative courses, this remains speculative and requires further study. From a practical standpoint, anesthesiologists should be aware of the potential for perioperative hyperexcitability in patients with anti-GAD antibody syndromes, consider benzodiazepine prophylaxis, and engage in multidisciplinary planning to optimize outcomes.
